# An international survey on retinopathy of prematurity practice patterns during the COVID-19 pandemic and lessons for future management

**DOI:** 10.1007/s10792-024-03290-8

**Published:** 2024-10-14

**Authors:** Rachel Shemesh, Michael Chiang, R. V. Paul Chan, Faruk Orge, Jason C. Yam, Sonal Farzavandi, Derek Sprunger, Eedy Mezer, Tamara Wygnanski-Jafee

**Affiliations:** 1 Faculty of Medicine, Tel-Aviv University, Tel-Aviv, Israel; 2 Goldschleger Eye Institute, Sheba Medical Center, 52621 Tel-Hashomer, Israel; 3 National Eye Institute, National Institutes of Health, Bethesda, USA; 4 National Library of Medicine, National Institutes of Health, Bethesda, MD USA; 5 Department of Ophthalmology and Visual Sciences, Illinois Eye and Ear Infirmary, University of Illinois, Chicago, USA; 6 Department of Ophthalmology and Visual Sciences, University Hospitals Rainbow Babies and Children’s Hospital, Case Western Reserve University, Cleveland, OH USA; 7 Department of Ophthalmology and Visual Sciences, The Chinese University of Hong Kong, Kowloon, Hong Kong China; 8 Singapore National Eye Centre, Singapore, Singapore; 9 Indiana University School of Medicine, Indiana, USA; 10 Department of Ophthalmology, Ruth Rappaport Children’s Hospital, Rambam Health Care Campus, Haifa, Israel; 11 Ruth and Bruce Rappaport Faculty of Medicine, Technion-Israel Institute of Technology, Haifa, Israel

**Keywords:** Retinopathy of prematurity, COVID-19 pandemic, Telemedicine, Retinal photography, Treatment modality

## Abstract

**Purpose:**

To assess retinopathy of prematurity (ROP) practice patterns during the coronavirus (COVID-19) pandemic.

**Methods:**

A survey on ROP practice patterns during the COVID-19 pandemic was sent to the American Academy of Ophthalmic Executives, the International Pediatric Ophthalmology and Strabismus Council members, and to various national societies on May 19, 2020. The survey closed on the 31st of June 2020.

**Results:**

Two hundred ninety-two ophthalmologists from 41 countries responded to the survey. Most replies originated in Asia (48%) and North America (38%). During the COVID-19 pandemic compared to the pre-COVID-19 period, respondents reported a reduction of 15% in the number of NICU inpatients and 19.8% of the ROP outpatients’ follow-up visits. The number of ROP outpatients’ follow-up visits and inpatients’ exams was significantly greater in North America than in Asia (72.0% versus 37.2% and 87.8% versus 49.6%, respectively, *P* < 0.001). Only 14% of the ophthalmologists adopted new screening guidelines, and 7.2% reported changing their preferred treatment. In 50% of responders, laser photocoagulation was the preferred treatment. A significantly higher percentage of ophthalmologists reported using telemedicine during the pandemic, 29.8% (n = 85/285), and 15.6% (44/282) prior to the pandemic (χ2 = 15.51, *p* < 0.001).

**Conclusions:**

During the COVID-19 pandemic, fewer ROP screening and follow-up visits were conducted on premature infants; these findings were especially prominent among physicians in Asia. Telemedicine usage increased during the pandemic. This study highlights the need to maintain screening protocols for ROP during pandemics. The utility of technological measures could enable this, along with adequate prevention of physical contact.

**Supplementary Information:**

The online version contains supplementary material available at 10.1007/s10792-024-03290-8.

## Introduction

On March 11, 2020, the World Health Organization (WHO) declared an outbreak of the coronavirus virus (COVID-19) a global pandemic [1]. Worldwide efforts to curb the spread of the virus have been made and many medical centers have issued guidelines as a pragmatic approach to maintain continuity of care for patients who need it, and deferred patients that can wait [2].

Retinopathy of prematurity (ROP) is a disorder of the developing retina of low-birth-weight preterm infants; it potentially leads to blindness in a small but significant percentage of infants [3]. Therefore, screening these infants is crucial to identify those who could benefit from treatment and to make appropriate recommendations regarding the timing of future screening; thus, it has been listed as an essential medical service during the COVID-19 pandemic by the American Academy of Ophthalmology (AAO) [4]. Katoch et al. reported a decrease in the number of infants screened for ROP, both as outpatients as well as in the neonatal intensive care unit (NICU) at a tertiary care institute in India during the COVID-19 lockdown (March–May 2020) [5]. However, a global perspective is needed in order to fully understand the changes that took place in screening and treating ROP during the pandemic. The purpose of this study was to assess the worldwide ROP practice patterns during the COVID-19 pandemic (May 2020).

## Methods

An email containing a weblink to a survey on ROP practice patterns during the COVID-19 pandemic (May 2020) was sent to ophthalmologists via the listserv to all American Academy of Ophthalmic Executives’ (AAOE) listservs and to International Pediatric Ophthalmology and Strabismus Council (IPOSC) members on May 19, 2020. The survey closed on the 31st of June 2020. The ROP practice patterns found in the survey data during COVID-19 are presented in Table 1. The questionnaire included 14 questions related to the characteristics of participants, practice patterns during the COVID-19 pandemic including follow-up visit volume, personal protection equipment (PPE), guidelines for ROP screening, treatment modalities, and usage of telemedicine. Each question was given a set number of answers from which the responders could choose the best fitting answer (Table 1). The number of participants, for the purpose of the statistical analysis, was determined by the number of responses received to a specific question in the questionnaire. The response was from an email address and could not be accepted more than once. Furthermore, we ascertained that each response was from a unique IP address. This study was approved by our regional institutional review board.

**Table 1:** The Questions outlined in the survey about Ophthalmologist’s’ global ROP practice patterns during the COVID-19 pandemic

No	Question
1	Where do you practice ophthalmology?
2	What kind of practice do you work in? a. Tertiary referral hospital b. Children’s hospital c. Other hospital setting d. Group practice (e.g., HMO) e. Solo private practice f. Other—Write In
3	What is the current number of your NICU follow-up visits compared to your pre-COVID-19 inpatient exam number? a. 0–25% b. 26–50% c. 51–75% d. 76–100% e. Over 100% I have temporarily stoped screening for ROP
4	What is the current number of your overall ROP outpatient follow-up visits compared to your pre-COVID-19 ROP outpatient exam? a. 0–25% b. 26–50% c. 51–75% d. 76–100% e. Over 100% I have temporarily stoped screening for ROP
5	Have you adopted newer guidelines that allow reduced ROP screening patterns compared to your pre-COVID-19 screening patterns? a. No b. Yes-write in
6	Has your preferred modality of treatment for ROP changed after the first wave of the COVID-19 pandemic? a. No b. Yes-write in
7	Which personal protection equipment (PPE) does the NICU require during your ROP exams? a. A surgical mask b. A N-95 or equivalent mask c. A face shield d. Glasses or eye goggles e. Hand gloves f. A gown g. Indirect mounted transparent shield h. No personal protection equipment i. Other-write in
8	What is your preferred treatment after the first wave of the COVID-19 pandemic? a. Combination of anti-VEGF and laser b. Intravitreal injection of an anti-VEGF agent > Laser photocoagulation of the peripheral retina c. Laser photocoagulation of the peripheral retina > Intravitreal injection of an anti-VEGF agent
9	Have you routinely used retinal photography for screening and follow-up of ROP pre-COVID-19? a. No b. Yes, how? write in
10	Are you currently using retinal photography after the first wave of the COVID-19 pandemic? a. No b. Yes, how? write in
11	Have you been using telemedicine with store-and-forward photography for screening and or follow-up ROP during the pre-COVID -19 period? a. No b. Yes
12	Do you now (after the first wave of the COVID-19 pandemic) prescribe dilating drops to the guardians so that the babies come pre-dilated to the outpatient ROP exam? a. No b. Yes
13	When a scheduled ROP exam during the COVID-19 pandemic is missed – what do you currently do? a. Reschedule a visit for the baby to be seen as soon as possible b. Reschedule a 1–2-week follow-up visit c. Ask to contact you once the pandemic restrictions have been lifted
14	Do you think there will be a rise in unfavorable outcomes regarding visual acuity and retinal anatomy for premature babies following the COVID-19 pandemic? a. No b. Yes, how? write in

*No* number, *COVID-19* coronavirus disease

### Statistical analysis

Categorical variables were described by their frequency and percentage. The chi-square test and Fisher’s exact test were used to compare the variables between North America and Asia; the Mann–Whitney test was used to compare the ordinal variables. All statistical tests were two sided and *p* < 0.05 was considered statistically significant. Statistical analysis was performed using SPSS statistical software (IBM SPSS Statistics, version 24, IBM Corp., Armonk, NY, USA, 2017).

## Results

### Demographics

**Fig. 1:**
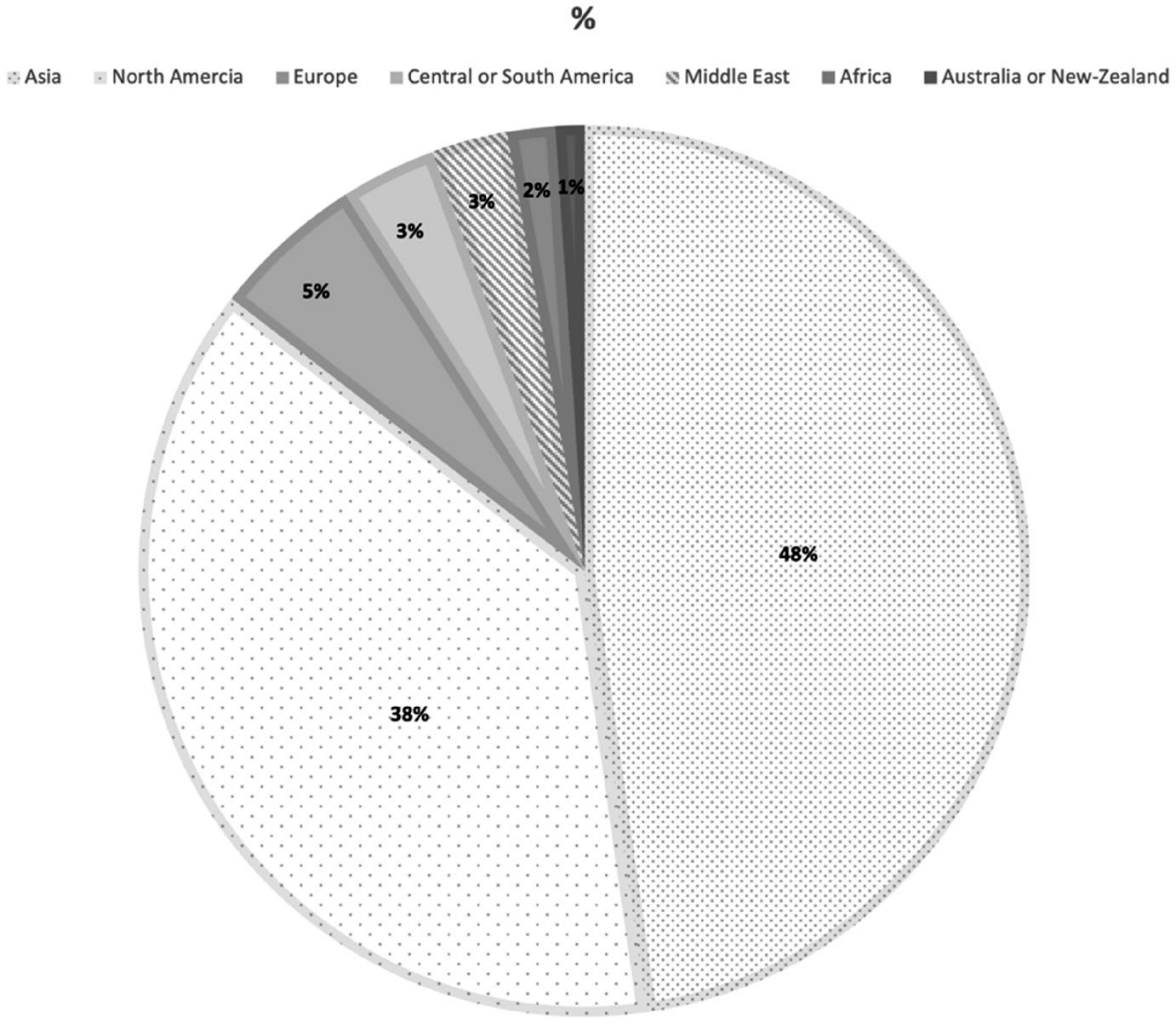
Geographical location of respondents during COVID-19 pandemic. This figure depicts the ophthalmologist’s geographical distribution

Two hundred and ninety-two ophthalmologists treating ROP from 226 different institutions around the world responded to the survey and 280 answered all the survey questions. Most ophthalmologists indicated that they practice medicine in Asia (47.8%, n = 138/289) or North America (37.7%, n = 109/289), and only 5.5% (n = 16/289) of them were located in Europe. The geographical distribution of ophthalmologists is presented in Fig. 1. Fifty percent of the ophthalmologists’ practice in a tertiary referral hospital (n = 146/292) and 32.5% in a children’s hospital (n = 95/292); 10% reported practicing in a group practice (n = 30/292) and 16.8% in a solo private practice (n = 49/292).

### Demographic differences between continents

A significantly higher number of physicians reported that they were working at a tertiary center in Asia (64.5%, n = 89/138) during the COVID-19 pandemic, compared with North America (28.4%, n = 31/109), (*p* < 0.001). In contrast, there were more physicians in North America working at a children’s hospital (41.3%, n = 45/109) and in a group practice (16.5%, n = 18/109), compared with Asia: 26.1%, n = 36/138 (*p* = 0.014), and 5.8%, n = 8/138 (*p* = 0.007), respectively.

### The outcomes of the ROP practice patterns

**Fig. 2:**
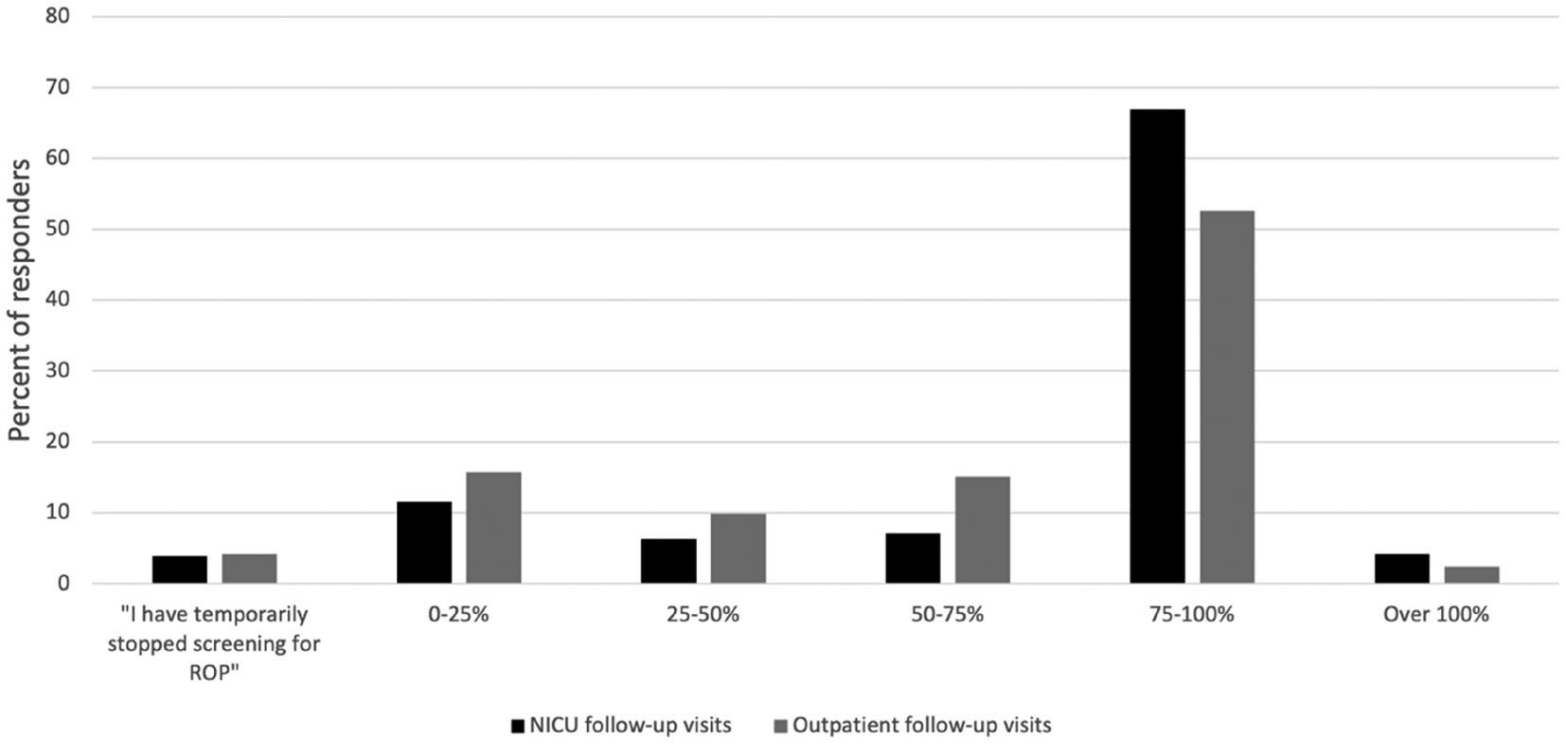
The change in volume of neonatal intensive care unit (NICU) and outpatient follow-up visits during COVID-19 pandemic compared to pre-Covid-19 exams. This figure depicts the change in volume of NICU and outpatient follow-up visits during COVID-19 pandemic compared to pre-Covid-19 exams

The majority of the respondents (66.9%, 190/284) stated that the current number of NICU follow-up visits is between 75 and 100%, compared to the pre-COVID-19 inpatient exams, and 11.6% (n = 33/284) reported that the current number is between 0 and 25% (Fig. 2). Over half of the respondents (52.6%, 150/285) reported that the current number of overall ROP outpatient follow-up visits is between 75 and 100%, compared to the pre-COVID-19 period, and 15.8% (n = 45/285) reported that the current number is between 0 and 25% (Fig. 2). Eighty-six percent (251/292) of the ophthalmologists did not adopt new screening guidelines during the COVID-19 pandemic (May 2020). The overwhelming majority (94.8%, 271/286) of the ophthalmologists did not change their ROP treatment routines and 92.6%, did not change their preferred treatment modality (n = 263/284). Most (71.2%, 208/292) ophthalmologists reported that a surgical mask was part of the PPE requirements during ROP exams at the NICU, 73.3% (n = 214/292) reported use of gloves, 42.5% (n = 124/292) reported use of gowns, and only 33.6% (n = 98/292) reported use of N-95 masks or equivalent masks as part of the PPE requirements. Half (50%, 10/20) of the respondents to the question “What is your preferred treatment after the first wave of the COVID-19 pandemic?” reported that laser photocoagulation was their preferred ROP treatment. A significantly higher percentage of ophthalmologists reported using telemedicine during the pandemic, 29.8% (n = 85/285), and 15.6% (44/282) prior to the pandemic (χ2 = 15.51, *p* < 0.001). Only 25.6% utilized routine retinal photography for screening during the pre-COVID -19 period (73/285), and 22% (n = 63/286) reported using retinal photography after the first wave of the COVID-19 pandemic (χ2 = 0.84, *p* = 0.36). When asked about missed scheduled ROP exams during the COVID-19 pandemic, most ophthalmologists (78.2%, 223/285) indicated that they would reschedule a visit for the infant as soon as possible, 16.8% (n = 48/285), and only 4.9% (n = 14/285) asked to be contacted once the pandemic restrictions have been lifted.

During the COVID-19 pandemic 29.8% (n = 85/285) indicated that they prescribe dilating drops to an infant’s parents or guardians, so that the infants arriving at the outpatient clinic are predilated for the ROP exam.

A total of 22.3% (65/292) of the respondents predicted that there would be an increased number of unfavorable outcomes for premature babies following the COVID-19 pandemic.

### Differences in ROP practice patterns between continents

The overall number of ROP outpatient follow-up visits, i.e., the percentage of physicians from a specific continent who monitored patients during the COVID-19 period, (North America: 72.0%, 77/107 versus Asia: 37.2%, 51/137) and inpatient exams in the NICU during the COVID-19 pandemic (North America: 87.8%, 93/106 versus Asia: 49.6%, 68/137) were significantly greater in North America than in Asia (*P* < 0.001 for both comparisons).

Significantly more physicians from Asia (8.8%, 12/137) indicated that they modified their ROP treatment during the pandemic in comparison to physicians from North America (1.9%, 2/108), (*p* = 0.025). There was no statistically significant difference between the percentage of physicians that adopted new guidelines that allow reduced ROP screening practice patterns in North America and in Asia (17.6%, 19/108) and (12.5%, 17/136) respectively, (*p* = 0.175). A significantly higher percentage of physicians responded that the surgical mask (51.9%, 94/138), N-95 or an equivalent mask (67.5%, /138), face shield (72.7%, 24/138), glasses or eye shield (66.3%, 53/138), and gowns (82.3%, 79/138) were part of their PPE during the pandemic in Asia, in comparison to physicians in North America with 48.1% using a surgical mask (87/109), (*p* = 0.043), 32.5% using N-95 or an equivalent mask (27/109), (*p* = 0.01), 27.3% using a face shield (9/109), (*p* = 0.039), 33.8% using glasses or eye shield (27/109), (p = 0.028), and 17.7% wearing gowns as part of their PPE (17/109), (*p* < 0.001). In addition, there was a significantly higher percentage of Asian respondents to the question “Have you routinely been using retinal photography for screening and in the follow-up of ROP pre-COVID-19?” (31.6%, 43/136), in comparison to their North American counterparts (15.7%, 17/108), (*p* = 0.003). A significantly higher percentage of physicians from North America indicated that they would reschedule a visit for the patients ASAP once a scheduled ROP follow-up exam is missed (86.0%, 98/137) in comparison to physicians from Asia (71.5%, 92/107), (*p* = 0.017).

## Discussion

ROP is a leading cause of preventable childhood blindness in middle-income countries [3]. In this study, we aimed at examining the management of this preventable disease during the COVID-19 pandemic. In this international survey on the ROP practice patterns during the COVID-19 pandemic, we found that during this period the number of ROP exams performed on premature infants decreased dramatically. A total of 11.6% of physicians reported that the number of inpatient exams in the NICU during COVID-19 was between 0 and 25%, compared to the pre-COVID-19 number (Fig. 2), and 15.8% reported that the number of outpatient follow-up visits during COVID-19 was between 0 and 25%, compared to the pre- COVID-19 number (Fig. 2). Katoch et al. described similar results in India [5]. Similar results were also found in other ophthalmology fields [6–8]. COVID-19 currently affects 219 countries, and all continents. A total of 292 ophthalmologists from 226 different institutions around the world participated in this survey (Fig. 1 and Table S1). Hence, the patterns seen in this study reflect the global ROP practice during this period. The decrease in ROP screening and treatment during COVID-19 could be attributed to several factors, including a shortage of health care staff [3, 9], transportation of teams deployed for general care [9] parents or guardians unwilling or unable to bring their infant to the hospital, and/or clinics being closed [10]. In addition, in this global study, we found that 23% of ophthalmologists examined less than 75% of inpatients in the NICU, compared with the pre-COVID-19 exams. This may indicate that in-house pediatric ophthalmologists were less available because physicians were deployed to other departments or ophthalmologists did not arrive due to the lockdown [11]. Furthermore, Ireland and Denmark reported that fewer premature babies were born in the spring of 2020. They indicated substantial reductions in the number of extremely preterm and very low birthweight births during this time [12, 13]. Our study also found a 47% reduction in the follow-up visits of outpatients during the pandemic. Other explanations for these findings, other than a lower rate of preterm births, include the parents or guardians being too afraid to come in for exams, as well as a higher rate of anxiety [14].

Only 14% of ophthalmologists adopted new guidelines that reduced the number of ROP screening patterns, compared to the pre-COVID-19 numbers, including switching to a risk-based follow-up, based on the Growth and the Retinopathy of Prematurity (G-ROP) guidelines [15], stopping follow-ups for immature zone 3 of low-risk patients, and changing the follow-up intervals of infants treated with VEGF injection to every 2 weeks instead of every week, which is the pre-COVID-19 follow-up pattern.

Only 7% of ophthalmologists reported changing their preferred treatment modality for ROP patients after the first wave of the COVID-19 pandemic (May 2020). The changes made included treating borderline cases by reducing the number of follow-up appointments and avoiding delayed treatment if a family member becomes ill with COVID-19. This finding is similar to the study of Leng et al. demonstrating that although a significant decrease in ophthalmology-related patient visit volume as a result of the COVID-19 pandemic was noted, it did not greatly impact age-related macular degeneration, diabetic macular edema, and macular edema secondary to retinal vein occlusion patients receiving intravitreal injections of anti-VEGF agents to stabilize and improve vision [16].

Unsurprisingly, 73% of ophthalmologists reported that surgical masks were part of their PPE requirements during ROP exams at the NICU, 74.1% reported gloves, and 43% reported that gowns were part of their requirements. These requirements were common in NICUs even prior to the COVID-19 pandemic. However, only 34% reported an N-95 mask or an equivalent mask as part of the PPE requirements. This could result from the limited global supply and competition for these masks; this led to severe shortages during the COVID-19 pandemic (May 2020), and it remains a major challenge and concern [17].

The benefits of anti-VEGF therapy, including the possibility of reduced permanent peripheral visual field loss, reduced anterior segment abnormalities, and less myopia [18] have made anti-VEGF therapy the preferred mode for many ROP patients. Interestingly, our results show that laser photocoagulation was still the preferred first mode of treatment during the COVID-19 pandemic and that the combination of laser photocoagulation and anti-VEGF treatment was used in 35% of the cases. Laser treatment of ROP has the advantage of fewer post-treatment visits; in contrast, anti-VEGF injections enable a shorter treatment session, with higher safety standards for both the infants and staff.

Traditionally, screening for ROP consists of an ophthalmologist examining the baby at the bedside using an indirect ophthalmoscope. In the last few years, telemedicine has been introduced as an alternative using remote interpretation of digital fundus images. Telemedicine enables mildly ill patients to receive the supportive care they need while minimizing their exposure to other acutely ill patients [19, 20]. This can explain the significant rise in telemedicine usage in May 2020 and emphasizes the importance of using technological measures to decrease the risk of exposure to contagious diseases during pandemics while maintaining screening protocols for ROP. Because retinal photography can be stored and forwarded to a remote expert for advice on the urgency of the referral, the use of these devices was expected to increase during the pandemic. Surprisingly, this did not happen. Sood et al. conducted a multicenter study in the US and found significantly more infants were screened with indirect ophthalmoscopy, compared to digital imaging, during the lockdown [21], similar to our study. This could result from several factors, including difficulty in purchasing cameras during the lockdown. In addition, using the equipment requires a learning curve; also, photography requires sterilization and may extend the exam duration, especially when initiated. Not using existing technologies could stem from the fact that this technological platform is less well known and unfamiliar to many ophthalmologists [22]. In addition, some physicians prefer using indirect ophthalmoscopy as it may still be required after using imaging due to the varying sensitivity of screening in cases of peripheral disease and media opacities. Another important factor could be the financial impact COVID-19 has caused on hospitals. While fewer patients were seen throughout every hospital system, in many cases, the health care workers were maintained on salary. With this increased burden, investing in relatively expensive retinal imaging systems and not being able to provide educational sessions on retinal imaging could have been important barriers to potentially increased demand.

In this study, the overall ROP outpatient follow-up visits and the number of inpatient visits at the NICU during the COVID-19 pandemic (May 2020) were significantly greater in North America, compared with the number of pre-COVID-19 ROP examinations in Asia. Most responders to our survey from Asia practiced medicine in India (Table S1). As India held the world’s longest lockdown starting in March 2020 until August 2020 [23] that left millions of people stranded in different parts of the nation with no means of transportation to reach clinics and hospitals [24, 25]. This could be the main reason for our finding. The pandemic first appeared in China, thus, the preparation for dealing with COVID-19 was very limited there and in neighboring countries [26]. This could also contribute to the lower outpatient and inpatient follow-up visits seen in our study. In contrast, in North America, lockdowns were not enforced, and hence follow up visits were possible, explaining the higher percentage of ROP exams.

This survey carries several limitations. Most importantly, the study included self-reported data from self-selected participants. The study was conducted in May 2020, a lockdown period in many countries, and this could be a significant constraint for people wishing to participate in the survey. Moreover, practice patterns may have changed in subsequent corona waves, which were not included in this study. In addition, the present study included only AAOE and IPOSC members that include only a small percentage of the population from each country, so caution should be exercised in generalizing the results to all ROP treating physicians globally. Furthermore, no data on screening numbers were collected, and the only estimate of the percentage change before and during the pandemic is subject to recollection bias. Longitudinal study of the important questions asked in this survey is needed, to fully understand the effect the COVID-19 pandemic had on ROP patients.

## Conclusions

During the COVID-19 pandemic (May 2020), fewer ROP screenings and follow-up visits were performed on premature infants; these findings were especially prominent among ophthalmologists in Asia. This could be attributed to lockdowns, a shortage of healthcare staff, transportation of teams deployed for general care, parents or guardians who were afraid, unwilling, or unable to bring their infant to the hospital, and a drop in premature births reported in the spring of 2020. A rise in telemedicine usage was seen in May 2020, emphasizing the importance of using technological measures to decrease the risk of exposure to contagious diseases during pandemics. Laser photocoagulation remained the preferred first mode of treatment. Twenty percent of ophthalmologists suspected unfavorable outcomes following the pandemic due to patients not being able to undergo retinal indentation. This international study highlights the need to maintain screening protocols for ROP during the COVID-19 pandemic. The utility of technological measures could enable this, along with adequate prevention of physical close contact.

## Supplementary Information

Below is the link to the electronic supplementary material.

Supplementary file1 (DOCX 16 kb)

## Data Availability

Data is provided within the manuscript or supplementary information files.
