# Supplementary material for: An international survey on retinopathy of prematurity practice patterns during the COVID-19 pandemic and lessons for future management
Source: Int Ophthalmol. 2024 Oct 14;44(1):407. doi: 10.1007/s10792-024-03290-8 (PMC11473615; doi:10.1007/s10792-024-03290-8)
Supplement: Supplementary file 1 — Supplementary file1 (DOCX 16 kb) [file 10792_2024_3290_MOESM1_ESM.docx]

Table S1 The continent and country distribution of respondents during COVID-19 pandemic

| **Continent** | **Country** | | **No. respondents for each country** | |  |  |
| --- | --- | --- | --- | --- | --- | --- |
| Asia | India | | 30 | |  |  |
|  | Singapore | | 17 | |  |  |
|  | Hong Kong | | 16 | |  |  |
|  | Japan | | 16 | |  |  |
|  | China | | 15 | |  |  |
|  | Philippines | | 11 | |  |  |
|  | Nepal | | 7 | |  |  |
|  | Malaysia | | 6 | |  |  |
|  | Indonesia | | 5 | |  |  |
|  | Bangladesh | | 4 | |  |  |
|  | Korea | | 2 | |  |  |
|  | Mongolia | | 1 | |  |  |
|  | Taiwan | | 4 | |  |  |
|  | Thailand | | 3 | |  |  |
|  | United Arab Emirates | | 1 | |  |  |
|  | Vietnam | | 1 | |  |  |
| North America | United States | | 108 | |  |  |
|  | Canada | | 3 | |  |  |
| Europe | United Kingdom | | 5 | |  |  |
|  | Germany | | 2 | |  |  |
|  | Netherlands | | 2 | |  |  |
|  | Spain | | 2 | |  |  |
|  | Denmark | | 1 | |  |  |
|  | Greece | | 1 | |  |  |
|  | Romania | | 1 | |  |  |
|  | Turkey | | 1 | |  |  |
|  | Ukraine | | 1 | |  |  |
| Central or South America | Costa Rica | | 4 | |  |  |
|  | Argentina | | 3 | |  |  |
|  | Chile | | 1 | |  |  |
|  | Ecuador | | 1 | |  |  |
|  | Paraguay | | 1 | |  |  |
| Middle East | Israel | | 5 | |  |  |
|  | Jordan | | 1 | |  |  |
|  | Qatar | | 1 | |  |  |
|  | Saudi Arabia | | 1 | |  |  |
| Australia | Australia | | 2 | |  |  |
| New Zealand | New Zealand | | 1 | |  |  |
| Africa | South Africa | | 3 | |  |  |
|  | Nigeria | | 1 | |  |  |
|  | Uganda | | 1 | |  |  |
|  | | |  | |  | |
|  | | |  | |  | |

No. - Number; COVID-19- Coronavirus disease
